# The Spine–Foot Connection: Investigating Compensatory Mechanisms in Degenerative Spine Disease Through Foot Deformity Patterns

**DOI:** 10.3390/medicina62071225

**Published:** 2026-06-24

**Authors:** Sereen Halayqeh, Austin Kaidi, Tomoyuki Asada, Quante Singleton, Dwayne Carney, Sheeraz Qureshi, Sravisht Iyer

**Affiliations:** 1 Department of Spine Surgery, Hospital for Special Surgery, New York, NY 10021, USA; halayqehs@hss.edu (S.H.); kaidia@hss.edu (A.K.); asadat@hss.edu (T.A.); singletonqu@hss.edu (Q.S.); carneyd@hss.edu (D.C.); qureshis@hss.edu (S.Q.); 2 Weill Cornell Medicine, New York, NY 10021, USA

**Keywords:** sagittal alignment, foot alignment, degenerative spine disease

## Abstract

*Background and Objectives*: In degenerative spine disease, compensatory mechanisms are activated to maintain upright posture, extending beyond the spine to involve the pelvis, lower limbs, and feet. These adaptations may be accompanied by differences in foot alignment, which could be associated with sagittal balance. The aim of this study is to investigate the relationship between foot alignment and spinal posture in patients with degenerative spine disease and evaluate whether foot deformities are associated with sagittal imbalance in degenerative spine disease. *Materials and Methods*: We retrospectively reviewed 98 patients with degenerative spine disease who underwent preoperative standing EOS imaging between 2017 and 2025 at a single academic spine centre. Meary’s angle, talocalcaneal angle, and calcaneal pitch were measured on lateral EOS images to classify feet as flat, normal, or cavus. Spinopelvic parameters were extracted from EOS and conventional radiographs. Differences in spinal parameters across foot groups were compared using ANOVA, and linear regression evaluated associations between sagittal vertical axis (SVA) and foot angles. *Results*: Among spinopelvic parameters, only SVA significantly differed between foot groups, with flatfoot patients showing greater forward imbalance (*p* = 0.035). Regression analysis demonstrated an inverse relationship between SVA and both talocalcaneal angle (*p* = 0.003) and calcaneal pitch (*p* = 0.034), suggesting that greater forward trunk inclination was associated with flatter feet. Degenerative scoliosis patients demonstrated a bimodal pattern with more flat and cavus feet (*p* = 0.006), while herniated disc patients more often exhibited flatfoot (*p* = 0.031). *Conclusions*: Foot posture abnormalities, particularly flatfoot, are associated with sagittal spinal imbalance, suggesting foot posture may be associated with global alignment and could reflect distal postural adaptations.

## 1. Introduction

Maintaining an upright posture with a horizontal gaze requires coordination of the spine, pelvis, and lower extremities, a concept described by Dubousset as the “cone of economy” and the “chain of balance” [1]. In degenerative spine disease, including degenerative disc disease, spinal stenosis, degenerative spondylolisthesis, and degenerative scoliosis, loss of lumbar lordosis or sagittal imbalance is associated with compensatory mechanisms—pelvic retroversion, knee flexion, and ankle dorsiflexion—to maintain the head over the feet [2,3]. The feet, as the distal link in this kinetic chain, also adapt: forward trunk shift moves the centre of gravity anteriorly and may be accompanied by ankle dorsiflexion and arch flattening (pes planus), which could help maintain balance. Conversely, a backward-leaning posture may be associated with a higher arch (pes cavus) [3].

Although pelvic and knee compensations are well described, the foot’s contribution to sagittal balance remains underexplored. A population study linked flatfoot to an increased risk of spinal degeneration, suggesting biomechanical interplay between the arch and spine [4]. However, whether spinal malalignment is associated with changes in foot posture or preexisting foot posture that influences spinal balance remains unclear.

This study aimed to examine the association between sagittal spinal alignment and foot posture in patients with degenerative spine disease using full-body EOS imaging. This study examined whether sagittal imbalance (higher SVA) would be associated with differences in foot alignment, reflecting the foot’s role in the global compensatory chain.

## 2. Materials and Methods

### 2.1. Study Design and Patient Selection

We conducted a retrospective cohort study of patients with degenerative spine conditions who underwent standing whole-body EOS radiography using the EOS imaging system (EOS imaging, Paris, France) at a single orthopedic hospital between July 2017 and June 2025. A total of 202 consecutive patients were screened. Inclusion criteria were age ≥ 18, diagnosis of degenerative spine disease, and availability of standing biplanar EOS images capturing the full spine and both feet. We excluded patients with prior major joint fusion or arthroplasty (hip/knee replacements) or foot/ankle surgery to avoid confounding from fixed alignment changes. Images with poor quality or positioning were also excluded. As a high-volume tertiary spine referral centre, our institution routinely evaluates patients with advanced degenerative pathology using standardized full-body EOS imaging. This population was appropriate for investigating global spinopelvic and foot alignment relationships, although findings may not fully generalize to community-based or non-surgical populations.

Baseline characteristics were recorded, along with preoperative patient-reported outcome measures (PROMs), including back and leg pain scores (VAS), Oswestry Disability Index (ODI), and PROMIS physical function T-scores.

Data collection and management were conducted using REDCap (Research Electronic Data Capture), hosted at the Weill Cornell Medicine Clinical and Translational Science Center. This platform is supported by the National Center for Advancing Translational Science of the National Institutes of Health under award number UL1 TR002384.

### 2.2. Radiographic Measurements

All measurements were obtained from standing EOS images. Sagittal spinal alignment parameters were measured using standard techniques on lateral images, including pelvic tilt (PT), pelvic incidence (PI), sacral slope (SS), lumbar lordosis (LL, L1–S1 Cobb angle), distal lumbar lordosis (L4–S1 segment lordosis), proximal lumbar lordosis (L1–L4 segment), PI–LL mismatch, maximum Cobb angle of lumbar scoliosis (measured on the AP view), and sagittal vertical axis (SVA).

For foot posture, three radiographic angles were measured on weight-bearing lateral EOS images of the feet. A trained researcher manually measured each angle using EOS PACS measurement tools, averaging the left and right sides when both were measurable, or using a single foot if only one side was imaged. Meary’s angle (talo-first metatarsal angle) was measured between the longitudinal axis of the talus and the first metatarsal; talocalcaneal angle (lateral tibiotalar-subtalar angle) was measured between the talus and calcaneus axes; and calcaneal pitch was measured as the angle between the inferior calcaneus and the horizontal floor.

These angles characterize foot arch height: a more negative Meary’s angle (talus pointing downward relative to the first metatarsal), a smaller talocalcaneal angle, or lower calcaneal pitch indicates flatfoot (pes planus), while a more positive Meary’s angle, larger talocalcaneal angle, or higher calcaneal pitch indicates a high arch (pes cavus). We classified foot deformities into flatfoot, normal, or cavus categories based on established thresholds [5,6,7]. For Meary’s angle, values < –2° were flatfoot, >6° were cavus, and –2° to 6° were normal. For the talocalcaneal angle, <44° was flatfoot, >54° was cavus, and 44–54° was normal. For calcaneal pitch, <20° indicated flatfoot, >26° indicated cavus, and 20–26° was normal. These groupings were applied separately to each measure to analyze foot posture by different definitions.

### 2.3. Data Analysis

Statistical analysis was carried out using IBM SPSS Statistics (version 29). We first performed descriptive analysis of the cohort’s baseline parameters. For comparisons of spinal alignment and clinical parameters between different foot posture groups, we used one-way analysis of variance (ANOVA). We performed these comparisons separately for groupings defined by Meary’s angle, talocalcaneal angle, and calcaneal pitch. Categorical data were analyzed with chi-square tests or Fisher’s exact tests as appropriate. Pearson’s correlation coefficients were calculated to examine bivariate associations between continuous spinal alignment metrics and foot angles. Finally, to identify independent predictors of foot posture, we constructed exploratory multivariable linear regression models with each foot angle as the dependent variable and a set of spinal alignment parameters as predictors. Statistical significance was set at *p* < 0.05 for all tests. No formal a priori sample size calculation was performed due to the exploratory nature of this study. The analysis was hypothesis-generating and intended to identify potential associations for future prospective investigation.

## 3. Results

### 3.1. Patient Characteristics

The final analysis included 98 patients with a mean age of 62.8 ± 14.8 years, 66% male, and a mean BMI of 27.6 ± 4.4 kg/m^2^. Common degenerative lumbar pathologies were present: 63% had lumbar central canal stenosis, 59% neuroforaminal stenosis, 48% degenerative disc disease, 24% degenerative spondylolisthesis, 17% herniated nucleus pulposus, and 9% degenerative lumbar scoliosis. Most patients (96%) were undergoing primary spine surgery. The average PI was 53.0° (±11.8), LL was −46.9° (±15.0), and PI–LL mismatch was +5.8° (±12.3). PT averaged 18.9° (±9.6) and mean SVA was 34.2 ± 38.2 mm. Disability was moderate (mean ODI 37%, VAS 5.4). Radiographic foot alignment indicated a tendency toward low arches: Meary’s angle was −1.9° ± 8.3, talocalcaneal angle was 37.6° ± 6.3, and calcaneal pitch was 16.2° ± 6.0, all suggesting common radiographic flatfoot deformities in this cohort (Table 1).

### 3.2. Prevalence of Flatfoot vs. Cavus

Depending on the measurement, a large majority of patients met criteria for flatfoot (pes planus). Using the talocalcaneal angle, 85–90% were classified as flatfoot (talocalcaneal < 44°), with very few (<5%) categorized as cavus, indicating that the “normal” or high-arch range was rare in this cohort. For many spine diagnoses, nearly all patients had talocalcaneal angles indicating flatfoot (Table 2). The calcaneal pitch classification also showed a high prevalence of low arches, with 70–80% of patients having a calcaneal pitch < 20° (flatfoot), and only 5–10% meeting cavus criteria. Meary’s angle showed a more balanced distribution: about half of the patients were classified as flatfoot (angle < −2°), 30–40% were normal, and 10–15% had cavus feet (Meary’s > 6°). All three measures consistently indicated that flatfoot deformity was more common than high arches, with the talocalcaneal angle being the most sensitive measure. However, the small “cavus” group in the talocalcaneal classification limited statistical power for between-group comparisons.

### 3.3. Associations by Spinal Diagnosis

We analyzed the relationship between spine diagnoses and foot posture (Table 2). Significant findings were seen in patients with herniated nucleus pulposus (HNP) and degenerative scoliosis. Among 17 HNP patients, 94% were classified as flatfoot by talocalcaneal angle (talocalcaneal < 44°), with no patients having normal, and only 6% cavus (*p* = 0.031). This association was less pronounced using other foot metrics, such as Meary’s angle (53% flatfoot vs. 41% normal, *p* = 0.677) and calcaneal pitch (82% flatfoot vs. 18% cavus, *p* = 0.286). For degenerative scoliosis (9 patients), Meary’s angle showed a bimodal distribution: 44% flatfoot, 44% cavus, and 11% normal (*p* = 0.006), indicating a tendency for either collapsed or high arches. The talocalcaneal and calcaneal pitch measures did not show significant associations in scoliosis. No other diagnoses showed significant differences in foot posture, as lumbar stenosis, spondylolisthesis, and degenerative disc disease all had high rates of flatfoot, but their distributions did not differ significantly from the overall cohort.

### 3.4. Correlation Analyses

**Figure 1:**
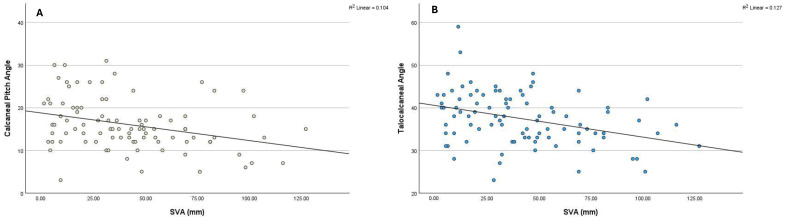
(**A**) Correlation between SVA and calcaneal pitch. (**B**) Correlation between SVA and talocalcaneal angle. SVA: sagittal vertical axis.

Bivariate Pearson correlations reinforced the group findings by quantifying continuous relationships between alignment and foot angles. Notably, SVA showed a moderate negative correlation with both talocalcaneal angle and calcaneal pitch (Table 3, Figure 1A,B). In other words, patients with larger forward imbalance (greater SVA) tended to have lower talocalcaneal angles and lower calcaneal pitch consistent with a flatter foot.

Interestingly, BMI was also significantly correlated with foot posture: higher BMI was associated with lower talocalcaneal angle and lower calcaneal pitch. Pelvic tilt showed no significant correlation with foot angles, nor did pelvic incidence or lumbar lordosis. Lastly, the clinical outcome scores (VAS, ODI, PROMIS) did not significantly correlate with foot angles.

### 3.5. Sagittal Alignment vs. Foot Posture

**Figure 2:**
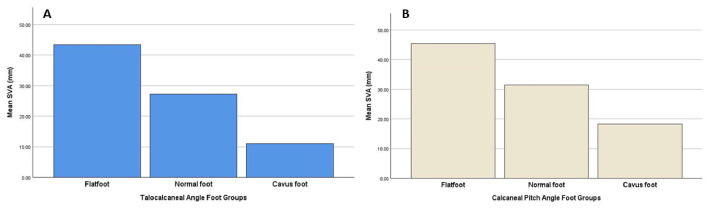
(**A**) SVA variation across foot deformity groups by talocalcaneal angle. (**B**) SVA variation across foot deformity groups by calcaneal pitch. SVA: sagittal vertical axis.

We examined whether sagittal spinal alignment differed among patients with flat, normal, or cavus feet. Figure 2A,B show the distribution of SVA across foot posture groups. SVA was significantly greater in patients with flatfoot alignment compared to those with cavus feet. For example, the flatfoot group (calcaneal pitch < 20°) had a mean SVA of 45.4 ± 29.6 mm, while the cavus group had 18.2 ± 13.7 mm, a more than two-fold difference. The talocalcaneal angle also showed a trend of higher SVA in flatfoot compared to cavus (mean SVA 43 mm vs. 11 mm), though this difference did not reach significance. No significant differences in other spinopelvic parameters or PROMs were observed among foot posture groups (Table 4A–C).

### 3.6. Multivariable Regression

To determine if spinal alignment parameters independently predicted the degree of foot deformity, we performed linear regressions for each foot-angle outcome (Table 5). In the model for the talocalcaneal angle, the only significant predictor was SVA. This indicates that greater forward imbalance is independently associated with a smaller talocalcaneal angle (flatter foot) even when controlling for pelvic parameters and lumbar curvature. Similarly, for the calcaneal pitch outcome, SVA emerged as a significant independent predictor, again indicating that higher SVA predicts a lower calcaneal pitch after adjusting for other alignment variables. Other spinopelvic parameters did not significantly influence foot angles in the multivariate setting.

## 4. Discussion

In this study, we investigated how degenerative spinal malalignment relates to foot deformities, and our findings provide new insight into how foot posture relates to global alignment in degenerative spine disease. To our knowledge, no prior study has evaluated the association between sagittal spinal alignment and radiographic foot posture using full-body EOS imaging in a degenerative spine population. Most prior literature has focused either on spinopelvic compensation or on epidemiologic associations between foot deformity and spinal degeneration, without quantifying sagittal alignment relationships. The key result was that SVA, a global measure of sagittal imbalance, differed significantly across foot posture types, with flatter feet associated with a more forward-shifted posture. In multivariate analysis, SVA was the only significant spinal predictor of foot angles, underscoring that global imbalance (rather than specific pelvic or lumbar angles) is strongly associated with foot posture changes, though directionality cannot be established. These findings extend the classic concept of sagittal compensation beyond the hips and knees, down to the foot level [2,3]. Our findings are consistent with the possibility that foot posture differences may accompany sagittal malalignment as part of the overall postural pattern. Importantly, this cross-sectional design does not allow determination of temporal sequence, and the observed associations may reflect shared biomechanical or demographic factors rather than a unidirectional compensatory mechanism.

Our results align with and build upon Dubousset’s “chain of balance” theory, which posits that the body acts as a linked system from the feet to the head [1]. Dubousset described how, in an ageing or pathologic spine, progressive compensations occur: lumbar lordosis is lost, the thoracic spine may flatten, the pelvis retroverts (increasing PT), and the knees flex, all to keep the head over the feet. What our study adds is quantitative evidence that the foot posture may be linked with the broader chain of postural alignment. Specifically, we observed that patients with significant sagittal imbalance often present with radiographic evidence of pes planus. One potential interpretation is that a flatter foot posture may permit greater tibial progression during stance. This is a plausible biomechanical explanation, though it cannot be confirmed in a cross-sectional study: a forward-shifted trunk would tend to push the centre of pressure toward the forefoot, and a flexible flatfoot lets the foot elongate and the ankle dorsiflex further, enabling the person to lean forward yet remain planted on the ground. Conversely, in some cases, limited ankle dorsiflexion, due to Achilles contracture, and a rigid foot structure, such as a stiff cavus deformity, may be associated with reduced available motion for distal postural adjustments.

Previous studies have indeed highlighted the ankle as a crucial yet “last resort” compensator in sagittal imbalance. Ouchida et al. found that in subjects with decompensated sagittal alignment (SVA ≥ 4 cm), knee flexion and ankle dorsiflexion were recruited in tandem, with ankle flexion showing an important role, especially in older patients [3]. Our findings are congruent with that: the strong correlation between SVA and foot arch angles suggests that ankle/foot compensation is engaged when sagittal imbalance becomes pronounced. In contrast, patients who maintained normal sagittal alignment (low SVA) in our cohort were not observed to have the same degree of arch flattening; some even had cavus feet, which could indicate a slight backward lean or simply a lack of anterior shift requiring compensation. While this mechanism is plausible, our cross-sectional data cannot determine whether the foot deformity arises as a result of spinal imbalance or whether pre-existing foot structure influences the observed compensation patterns.

It is worth noting that while PT is classically the first compensatory mechanism, pelvic retroversion increases as lordosis decreases [3]. We did not find a direct correlation between PT and foot posture. This might be because PT primarily compensates in earlier stages of imbalance, and only when pelvic retroversion reaches its limit (due to anatomical constraints of hip extension) do patients recruit distal joints [2]. At that point, knee flexion and ankle dorsiflexion kick in rapidly. By the time a patient shows overt forward stooping (high SVA), they may have already maximized PT, and the flatfoot posture may be one of several distal features observed in patients with greater forward alignment to stay upright. This progression is supported by prior work; Diebo et al. reported that as PI–LL mismatch worsens, the contributions of pelvic tilt and thoracic curvature compensation diminish, while knee flexion and ankle dorsiflexion contributions increase [8]. Thus, our data reinforces the idea that foot alignment changes (flattening) may be associated with more pronounced sagittal imbalance.

An unexpected finding in our study was the different patterns of foot posture observed in specific spinal conditions, particularly herniated disc vs. degenerative scoliosis. Nearly all patients with lumbar disc herniation (HNP) had flat feet by our talocalcaneal angle criterion. Why might a lumbar disc prolapse be associated with pes planus? One possibility is that HNP patients were generally younger (our HNP subset mean age was lower) and had higher BMI, factors known to predispose to flexible flatfoot. It is known that obesity and younger ligamentous laxity can lead to planovalgus foot deformity, and indeed, we found BMI correlated with flatter feet. Another explanation is that patients with acute disc herniation and radicular pain often adopt a flexed-forward posture (antalgic forward flexion to relieve nerve tension) [2]. This forward-flexed stance may be accompanied by increased ankle dorsiflexion and pronation. Additionally, an L5 or S1 nerve root compression in HNP could cause subtle weakness of the calf or intrinsic foot muscles, potentially reducing the dynamic support of the arch (for example, tibialis posterior is an L5-S1 innervated muscle crucial for arch support). While speculative, it is conceivable that neural factors could be considered as a possible contributor, though this remains speculative. On the other hand, degenerative scoliosis patients in our study showed a split: some had very flat feet, others very high arches. One interpretation is that degenerative scoliosis, which often has a lateral imbalance component, may be associated with asymmetric loading: perhaps one foot becomes pronated (on the convex side) and the other supinated (concave side). Since we averaged or categorized feet together in this analysis, a patient with one flat and one cavus foot might appear as having both, contributing to the bimodal distribution. Another factor is that degenerative scoliosis cases may include concomitant neurological changes (spinal cord or nerve compression) that can influence muscle tone in the legs and feet, sometimes leading to cavus foot (for instance, subtle neuropathy or muscle imbalance can manifest as cavus). Notably, a prior study has drawn connections between neuromuscular scoliosis and cavus foot deformity, as seen in Charcot–Marie–Tooth disease [9], but in degenerative scoliosis, the mechanism is less clear. Our finding of a significant association on Meary’s angle for scoliosis (nearly half cavus) warrants further investigation; it suggests that spinal coronal deformity or spinopelvic asymmetry may be reflected in foot posture, possibly through compensatory shifting of weight or underlying patient factors. Clinically, this means spine specialists should be aware that a patient with degenerative scoliosis might present with either end of the foot deformity spectrum, and a one-size-fits-all expectation (“all scoliosis patients have flatfoot”) would be incorrect.

When comparing our results with the existing literature, we found generally supportive evidence, but also one contrasting report. A 2019 conference abstract by Khan et al. claimed, “no evidence that foot radiographs predict sagittal alignment or degeneration of the spine” [10]. However, that study seemed to focus on foot deformity as a predictor of having spinal degeneration more akin to the epidemiologic question addressed by Chou et al. [4], whereas our focus was on the degree of imbalance correlating with foot posture within a population already having spinal issues. The cohort differences (general versus spine patients) and methodology could explain the discrepancy. On the whole, our findings actually harmonize with the epidemiologic studies; for example, Chou et al. observed that flatfoot patients had a higher incidence of spondylosis and disc disorders [4]. This could be interpreted in reverse: perhaps those with spinal degeneration (like our patients) tend to manifest flatfoot, consistent with our cohort, where flatfoot was very prevalent. Similarly, Hsu et al. showed hallux valgus is associated with spinal degenerative disease (adjusted OR 1.7), further underscoring that foot deformities and spinal pathology often coexist [11]. Our study adds the nuance that, beyond co-existence, there appears to be a functional link: the severity of sagittal malalignment correlates with the severity of arch collapse.

From a clinical perspective, these findings highlight the importance of a holistic evaluation of posture in degenerative spine patients. Surgeons and clinicians should be aware that a patient stooping forward due to lumbar degeneration may also present with flatfoot deformity as part of their adaptation. This has practical implications. For instance, when planning corrective spine surgery, one might consider how the lower-extremity alignment may change following sagittal realignment, although this study did not assess postoperative posture. If a patient has fixed contractures in the hips or knees, or a rigid foot deformity, those could limit their ability to stand upright even after spine realignment [3]. If a flatfoot is severe and rigid, it might merit concurrent attention (e.g., use of orthotics or referral to a foot and ankle specialist), especially if it affects the overall postoperative posture or gait. Conversely, a cavus foot can indicate a different issue, possibly a neurological component, and might prompt an exam for underlying neuropathy or imbalance. Additionally, our observation that foot posture did not correlate with ODI or pain suggests that many patients may not complain about the foot at all. The foot change is “silently” helping them compensate. But removing that compensation, for example, via surgery correcting the spine, could unmask issues like gastrocnemius tightness or foot strain once the patient’s alignment shifts. Thus, a comprehensive approach to sagittal alignment should extend to checking ankle range of motion and foot alignment.

Our study also suggests that different degenerative diagnoses utilize different compensation patterns. A practical example is a patient with a large disc herniation: they are often leaning forward (positive sagittal balance) to alleviate nerve pain [2], and our data suggest they more frequently demonstrate radiographic features consistent with flatter foot posture. Treating the disc herniation (surgically or conservatively) may allow them to stand more erect, which could change how their foot loads. In degenerative scoliosis patients, careful assessment of balance in both sagittal and coronal planes is needed; a cavus foot in a scoliosis patient might indicate they are offloading to one side or have a long-standing muscle imbalance. Recognizing these patterns can improve individualized care. It also raises interesting questions for future research, for example, do patients with flatfoot compensation have different outcomes after spine surgery compared to those who do not require foot compensation? Does correcting a flatfoot (with orthotics or surgery) improve balance or pain in spine patients? These remain to be studied.

This study has several limitations. First, it was a retrospective, cross-sectional analysis conducted at a single tertiary spine referral centre, which may introduce referral bias and limit generalizability. Our cohort consisted of patients undergoing preoperative evaluation with EOS imaging, likely representing individuals with more advanced or symptomatic degenerative pathology. Therefore, the findings may not generalize to non-surgical, community-based, or asymptomatic populations. The high prevalence of radiographic flatfoot observed in this cohort may reflect characteristics of this referral population and should be interpreted cautiously.

Second, the cross-sectional design precludes the determination of temporal sequence or causality. We cannot establish whether sagittal malalignment is associated with adaptive changes in foot posture, whether pre-existing foot deformities influence spinal alignment, or whether both are influenced by shared factors such as age, BMI, or neuromuscular characteristics. The findings should therefore be considered associative and hypothesis-generating.

Third, foot deformity is inherently three-dimensional, yet our assessment relied exclusively on lateral radiographic parameters. Coronal-plane alignment, forefoot abduction, and rotational components were not evaluated, potentially leading to incomplete characterization of complex deformities. In addition, left and right foot measurements were averaged when both were available, which may have masked clinically relevant asymmetries, particularly in patients with coronal spinal deformity such as degenerative scoliosis.

Fourth, the flexibility of foot deformities was not assessed. We could not distinguish between flexible (postural) and rigid (structural) deformities, limiting the interpretation of whether observed patterns represent dynamic postural adaptations or fixed structural alignment.

Fifth, although exploratory multivariable models were performed, residual confounding remains possible. BMI was correlated with foot posture and may plausibly relate to sagittal alignment parameters; however, the study was not powered for comprehensive confounder adjustment. Additionally, no formal a priori sample size calculation was performed, and multiple comparisons were conducted without correction, increasing the risk of type I error.

Finally, some subgroup analyses were limited by small and imbalanced sample sizes, particularly in degenerative scoliosis and cavus foot groups. These findings should be interpreted cautiously and viewed as exploratory rather than definitive.

Prospective, multi-centre studies incorporating dynamic assessment, comprehensive multi-planar foot evaluation, and longitudinal follow-up are needed to clarify these relationships.

## 5. Conclusions

Sagittal alignment, particularly SVA, was associated with foot posture in patients with degenerative spine disease. Flatfoot alignment was more frequently observed in patients with greater forward imbalance. Foot posture was associated with sagittal alignment measures and may reflect distal features of global postural alignment; causality cannot be determined from this cross-sectional analysis.

## Figures and Tables

**Table 1:** Patient demographics, diagnoses, and baseline clinical characteristics.

Variable	Mean/Count	SD/%
**Demographics**		
Age (years)	62.8	14.8
Body Mass Index (kg/m^2^)	27.6	4.4
Gender		
Male	65	66.3%
Female	33	33.7%
**Spine Diagnoses**		
Degenerative Disc Disease/Spondylosis	47	48.0%
Herniated Nucleus Pulposus	17	17.3%
Central Stenosis	62	63.3%
Neuroforaminal Stenosis	58	59.2%
Lateral Recess Stenosis	51	52.0%
Degenerative Spondylolisthesis	23	23.5%
Isthmic Spondylolisthesis	5	5.1%
Radiculopathy	36	36.7%
Degenerative Scoliosis	9	9.2%
**Surgical Data**		
Primary Surgery	94	95.9%
Revision Surgery	4	4.1%
**Patient-Reported Outcomes (PROMs)**		
Back VAS (0–10)	5.5	3.0
Leg VAS (0–10)	5.4	3.0
ODI (%)	37.4	16.9
PROMIS Score	36.4	6.8
**Spinal Alignment Parameters (°/mm)**		
Pelvic Tilt (PT)	18.9	9.6
Sacral Slope (SS)	32.3	11.2
PI–LL Mismatch	5.8	12.3
Sagittal Vertical Axis (SVA, mm)	34.2	38.2
Distal Lumbar Lordosis	–27.7	12.6
Max Lumbar Cobb Angle	–2.9	14.0
**Foot Alignment Parameters (°)**		
Meary’s Angle	–1.9	8.3
Talocalcaneal Angle	37.6	6.3
Calcaneal Pitch	16.2	6.0

**Table 2:** Distribution of spine diagnoses across foot deformity groups based on Meary’s angle, talocalcaneal angle, and calcaneal pitch.

Spine Diagnosis	Meary’s Angle			*p*-Value	Talocalcaneal Angle			*p*-Value	Calcaneal Pitch			*p*-Value
	Flatfoot	Normal Foot	Cavus Foot		Flatfoot	Normal Foot	Cavus Foot		Flatfoot	Normal Foot	Cavus Foot	
Degenerative Disc Disease/Spondylosis	20 (42.6%)	20 (42.6%)	7 (14.9%)	0.355	41 (87.2%)	5 (10.6%)	1 (2.1%)	0.570	34 (72.3%)	8 (17.0%)	5 (10.6%)	0.187
Herniated Nucleus Pulposus	9 (52.9%)	7 (41.2%)	1 (5.9%)	0.677	16 (94.1%)	0 (0.0%)	1 (5.9%)	0.031 *	14 (82.4%)	1 (5.9%)	2 (11.8%)	0.286
Central Stenosis	32 (51.6%)	22 (35.5%)	8 (12.9%)	0.828	53 (85.5%)	9 (14.5%)	0 (0.0%)	0.082	49 (79.0%)	11 (17.7%)	2 (3.2%)	0.277
Neuroforaminal Stenosis	28 (48.3%)	22 (37.9%)	8 (13.8%)	0.834	52 (89.7%)	5 (8.6%)	1 (1.7%)	0.591	45 (77.6%)	9 (15.5%)	4 (6.9%)	0.907
Lateral recess stenosis	23 (45.1%)	19 (37.3%)	9 (17.6%)	0.217	43 (84.3%)	8 (15.7%)	0 (0.0%)	0.108	38 (74.5%)	10 (19.6%)	3 (5.9%)	0.658
Degenerative Spondylolisthesis	9 (39.1%)	9 (39.1%)	5 (21.7%)	0.230	21 (91.3%)	2 (8.7%)	0 (0.0%)	0.821	15 (65.2%)	5 (21.7%)	3 (13.0%)	0.176
Isthmic Spondylolisthesis	2 (40.0%)	3 (60.0%)	0 (0.0%)	0.486	5 (100.0%)	0 (0.0%)	0 (0.0%)	0.717	5 (100.0%)	0 (0.0%)	0 (0.0%)	0.466
Radiculopathy	19 (52.8%)	10 (27.8%)	7 (19.4%)	0.136	33 (91.7%)	3 (8.3%)	0 (0.0%)	0.661	27 (75.0%)	5 (13.9%)	4 (11.1%)	0.277
Degenerative Scoliosis	4 (44.4%)	1 (11.1%)	4 (44.4%)	0.006 *	9 (100.0%)	0 (0.0%)	0 (0.0%)	0.534	8 (88.9%)	1 (11.1%)	0 (0.0%)	0.626

Bold values and * indicate statistically significant *p*-values (*p* < 0.05).

**Table 3:** Pearson correlation between spinal parameters and foot angles.

Variables	Correlation	Meary’s Angle	Talocalcaneal Angle	Calcaneal Pitch
**Age**	Pearson Correlation	−0.199	−0.050	−0.144
	*p*-value	0.050	0.623	0.158
**BMI**	Pearson Correlation	−0.076	−0.318	−0.314
	*p*-value	0.455	0.001 *	0.002 *
**PT**	Pearson Correlation	0.101	−0.157	−0.036
	*p*-value	0.330	0.128	0.728
**SS**	Pearson Correlation	0.110	−0.017	0.128
	*p*-value	0.284	0.870	0.213
**PI**	Pearson Correlation	0.176	−0.107	0.061
	*p*-value	0.087	0.300	0.558
**LL**	Pearson Correlation	0.134	0.044	0.172
	*p*-value	0.193	0.667	0.094
**PI-LL**	Pearson Correlation	0.076	−0.179	−0.137
	*p*-value	0.463	0.081	0.184
**SVA**	Pearson Correlation	−0.016	−0.345	−0.329
	*p*-value	0.884	<0.001 *	0.001 *
**Distal LL**	Pearson Correlation	−0.001	−0.048	−0.098
	*p*-value	0.991	0.647	0.349
**Proximal LL**	Pearson Correlation	−0.113	0.031	−0.082
	*p*-value	0.278	0.766	0.434
**Maximum Lumbar Cobb**	Pearson Correlation	0.196	−0.182	−0.007
	*p*-value	0.078	0.102	0.952
**Back VAS**	Pearson Correlation	0.086	−0.001	0.109
	*p*-value	0.406	0.990	0.289
**Leg VAS**	Pearson Correlation	−0.050	0.033	0.008
	*p*-value	0.629	0.747	0.941
**ODI**	Pearson Correlation	0.061	−0.130	−0.015
	*p*-value	0.557	0.210	0.888
**PROMIS**	Pearson Correlation	0.046	0.055	0.048
	*p*-value	0.661	0.602	0.647

Bold values and * indicate statistically significant *p*-values (*p* < 0.05).

**Table 4:** A. Spinal measures and PROMs across Meary’s angle foot groups; B. spinal measures and PROMs across talocalcaneal angle foot groups; C. spinal measures and PROMs across calcaneal pitch foot groups.

Variable	Flatfoot (M ± SD)	Normal Foot (M ± SD)	Cavus Foot (M ± SD)	*p*-Value
A				
PT (°)	17.68 ± 11.45	19.59 ± 7.38	21.38 ± 7.89	0.424
SS (°)	31.02 ± 11.95	34.04 ± 10.54	32.33 ± 10.52	0.478
PI (°)	50.42 ± 13.20	53.79 ± 10.62	53.48 ± 9.35	0.398
LL (°)	–44.90 ± 15.45	–47.85 ± 15.94	–46.66 ± 12.82	0.682
PI–LL mismatch (°)	5.52 ± 13.09	5.94 ± 12.60	6.82 ± 8.37	0.947
SVA (mm)	33.09 ± 36.14	36.97 ± 40.37	30.26 ± 42.40	0.847
Distal LL (°)	–27.73 ± 14.00	–28.34 ± 11.85	–25.84 ± 10.20	0.841
Proximal LL (°)	–17.34 ± 9.86	–19.51 ± 11.10	–20.82 ± 8.53	0.467
Lumbar Cobb (°)	–4.21 ± 12.97	–3.65 ± 12.08	5.00 ± 21.47	0.165
Back VAS	5.32 ± 3.11	5.45 ± 2.97	6.50 ± 2.28	0.465
Leg VAS	5.78 ± 2.90	4.74 ± 2.76	5.67 ± 3.89	0.274
ODI	36.64 ± 18.24	37.19 ± 14.92	41.15 ± 17.77	0.712
PROMIS	36.74 ± 7.67	35.86 ± 5.59	36.92 ± 6.72	0.822
B				
PT (°)	18.85 ± 9.69	19.56 ± 10.28	15.00	0.904
SS (°)	32.32 ± 11.64	32.22 ± 7.48	36.00	0.949
PI (°)	52.13 ± 12.08	51.89 ± 10.60	51.00	0.994
LL (°)	–45.65 ± 15.35	–51.78 ± 14.95	–49.00	0.515
PI–LL mismatch (°)	6.49 ± 11.85	0.11 ± 16.21	2.00	0.322
SVA (mm)	43.39 ± 30.53	27.13 ± 17.13	11.00	0.202
Distal LL (°)	–27.08 ± 12.73	–33.89 ± 10.49	–	0.125
Proximal LL (°)	–18.72 ± 10.28	–17.89 ± 9.99	–	0.818
Lumbar Cobb (°)	–2.16 ± 13.94	–10.51 ± 13.60	–	0.133
Back VAS	5.48 ± 3.00	5.60 ± 2.77	8.00	0.700
Leg VAS	5.37 ± 2.99	5.30 ± 3.29	6.00	0.976
ODI	37.31 ± 16.95	36.91 ± 17.38	52.00	0.689
PROMIS	36.59 ± 6.57	35.86 ± 8.75	29.60	0.574
C				
PT (°)	19.17 ± 9.94	17.94 ± 9.79	17.83 ± 5.64	0.868
SS (°)	31.37 ± 11.41	36.69 ± 10.53	32.83 ± 9.28	0.228
PI (°)	51.62 ± 12.06	54.88 ± 12.38	50.67 ± 6.80	0.584
LL (°)	–44.75 ± 15.02	–52.94 ± 16.11	–47.00 ± 13.22	0.150
PI–LL mismatch (°)	6.86 ± 11.92	1.94 ± 15.14	3.67 ± 6.59	0.318
SVA (mm)	45.38 ± 29.61	31.53 ± 30.65	18.20 ± 13.70	0.021 *
Distal LL (°)	–26.98 ± 13.11	–32.88 ± 9.89	–22.20 ± 10.28	0.145
Proximal LL (°)	–17.93 ± 10.71	–20.06 ± 8.25	–24.40 ± 6.54	0.327
Lumbar Cobb (°)	–2.39 ± 14.16	–5.21 ± 15.34	–3.73 ± 8.71	0.813
Back VAS	5.36 ± 3.14	5.56 ± 2.16	7.33 ± 2.07	0.293
Leg VAS	5.45 ± 3.00	5.13 ± 2.63	5.00 ± 4.15	0.886
ODI	37.47 ± 16.83	34.23 ± 14.26	45.33 ± 23.65	0.392
PROMIS	36.45 ± 6.65	36.91 ± 7.74	35.03 ± 6.65	0.849

Bold values and * indicate statistically significant *p*-values (*p* < 0.05).

**Table 5:** Multivariable linear regression analyses predicting foot angles from spinal parameters.

Predictors	Outcome/Dependent	β	*t*-Value	*p*-Value	95% CI		R^2^
					Lower	Upper	
PT	Talocalcaneal angle	−0.140	−0.084	0.934	−1.985	1.825	0.202
SS		−0.064	−0.034	0.973	−1.943	1.878	
PI		−0.166	−0.059	0.953	−2.791	2.629	
PI–LL		0.132	0.029	0.977	−4.523	4.659	
SVA		−0.372	−3.101	0.003 *	−0.115	−0.025	
Distal LL		−0.249	−0.048	0.962	−4.699	4.477	
Proximal LL		−0.110	−0.029	0.977	−4.649	4.516	
Max Lumbar Cobb		0.117	0.996	0.323	−0.067	0.202	
PT	Calcaneal pitch angle	−0.691	−0.389	0.699	−2.485	1.674	0.103
SS		−0.791	−0.393	0.696	−2.497	1.675	
PI		−1.542	−0.522	0.603	−3.733	2.184	
PI–LL		2.132	0.448	0.655	−3.885	6.139	
SVA		−0.275	−2.167	0.034 *	−0.103	−0.004	
Distal LL		−2.507	−0.458	0.648	−6.159	3.858	
Proximal LL		−1.898	−0.473	0.638	−6.188	3.817	
Max Lumbar Cobb		0.039	0.314	0.754	−0.124	0.170	

Bold values and * indicate statistical significance (*p* < 0.05).

## Data Availability

The data presented in this study are not available to the public but would be available upon reasonable request.
